# Leiomyosarcoma with osteoclast‐like (LMS‐OGC) giant cells the breast: A report of a rare case

**DOI:** 10.1111/1759-7714.13190

**Published:** 2019-09-01

**Authors:** Zhonghai Xu, Junjun Gu, Shan Zhang, Zhanjiao Zhang, Wei Fang

**Affiliations:** ^1^ Department of Pathology, Kingmed Diagnostics Nanjing Jiangsu Province China; ^2^ Department of Pathology Traditional Chinese Medicine Hospital of Dafeng City Yancheng Jiangsu Province China; ^3^ Department of Emergency Zhuji People's Hospital of Zhejiang Province Zhuji Zhejiang Province China

**Keywords:** Breast, leiomyosarcoma, osteoclast‐like giant cells

## Abstract

Leiomyosarcoma with osteoclast‐like giant cells (LMS‐OGC) has seldom been reported clinically. LMS‐OGC of the breast is extremely rare according to the literature. Here, we report a case of LMS‐OGC leiomyosarcoma with osteoclast‐like giant cells of the breast. A 51‐year‐old female patient presented with a breast mass which was treated surgically and the pathological examination of the tumor indicated LMS‐OGC. Microscopically, the tumor was composed of spindle cells arranged in bundles or spokes with giant tumor cells and mitosis. Eosinophilic cytoplasm and morphologically benign osteoclast‐like cells were mixed together. Immunohistochemistry examination revealed SMA and desmin were positive with a Ki‐67 proliferation index of 40%. However, CK (AE1/AE3), E‐cadherin, ER, PR, CD34, S‐100 and CD10 were negatively expressed in the tumor tissue. LMS‐OGC is a soft tissue malignant tumor which develops extremely rarely in the breast. It should be differentiated and diagnosed according to the history, histological characteristics and immunohistochemical staining.

## Introduction

Leiomyosarcoma with osteoclast‐like giant cells (LMS‐OGC) is a rare and special type of leiomyosarcoma first reported by Darby *et al*.^1^ Several studies have previously reported LMS‐OGC in the uterus.^1^, ^2^ However, LMS‐OGC developed from the breast is extremely rare and only one case has been reported according to the literature.^3^ Here, we present a very rare case of LMS‐OGC arising from the breast which makes an important contribution to the literature.

## Case report

**Figure 1:**
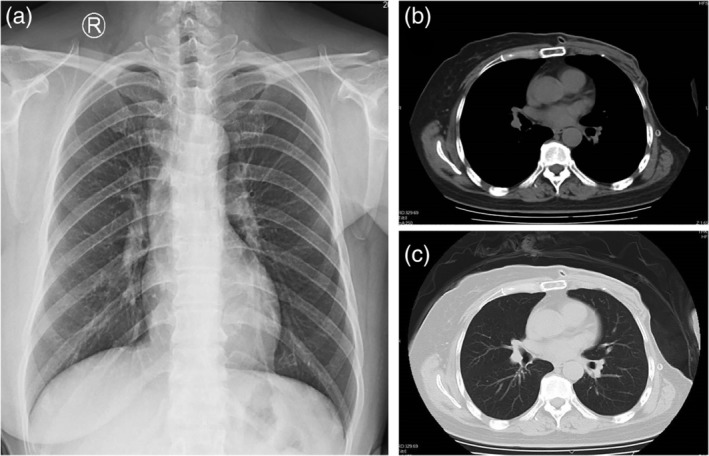
Digital radiography (D) and computed tomographic (CT) examination of the chest after surgery (**a**) DR of the chest, (**b**) Mediastinal window view of the breast and (**c**) Lung window view of the breast.

**Figure 2:**
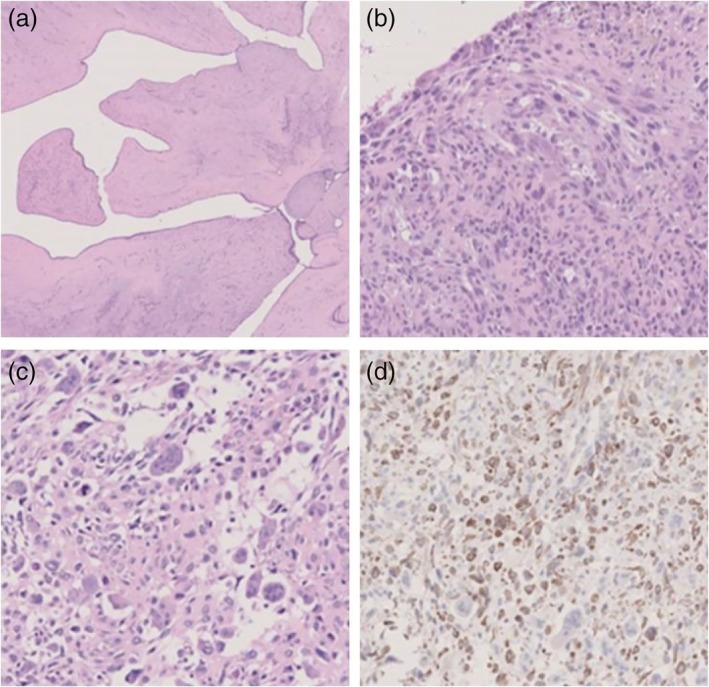
Pathology and immunohistochemistry examination of the LMS‐OGC (**a**) Lobular neoplasm structure area of the tumor, 2.5×, (**b**) Interstitial cells were obviously proliferated and the cytoplasm of interstitial cells was eosinophilic, 10×, (**c**) Giant tumor cells, 10 and (**d**) desmin positive expression 10×).

A 51‐year‐old female patient presented with a breast mass which was treated by surgery in December 2017 in the Traditional Chinese Medicine Hospital of Dafeng City (Fig 1). The tumor mass was subsequently sent to the Department of Pathology, Kingmed Clinical Laboratory Co., Ltd. for pathology consultation and was diagnosed as leiomyosarcoma with osteoclast‐like giant cells (LMS‐OGC). After nine months follow‐up, the tumor had recurred and surgical excision of the mass was again performed. Pathological examination confirmed this was LMS‐OGC of the breast. Microscopically, the tumor were composed of spindle cells arranged in bundles or spokes with giant tumor cells and mitosis (Fig 2). Eosinophilic cytoplasm and morphologically benign osteoclast‐like cells were mixed together. Immunohistochemistry examination revealed the SMA and desmin were positive with a Ki‐67 proliferation index of 40%. However, CK (AE1/AE3), E‐cadherin, ER, PR, CD34, S‐100 and CD10 were negatively expressed in the tumor tissue.

## Discussion

Osteoclast‐like giant cells are found in a variety of tumors, most commonly in bone tumors, followed by epithelial tumors, and a few in soft tissue tumors. The origin of OGC in LMS‐OGC is controversial with different opinions including that it originates from the reactive mesenchymal cells in the tumorigenesis process, or from undifferentiated pluripotent stem cells. The mechanism of OGC generation and aggregation in tumors is speculative. Although its clinical significance is not clear, it is an important clue for pathological diagnosis and a reliable basis for prognostic evaluation.

LMS‐OGC of breast should be differentiated from the following tumors: (i) Giant cell malignant fibrous histiocytoma (MFH) also known as undifferentiated pleomorphic sarcoma with giant cells. The main difference between giant cell MFH and LMS‐OGC is that there is no classical smooth muscle tumor area in giant cell MFH. In addition, immunohistochemical staining is also helpful for identification. Giant cell MFH does not express SMA, desmin and other smooth muscle markers^4^ (ii) Mammary carcinoma with osteoclast‐like giant cells can present as mucinous carcinoma, invasive lobular carcinoma, squamous cell carcinoma and other forms. On this basis, multinucleated giant cells appear in the stroma. The giant cells positively express epithelial markers of EMA (+) and CK (+). (iii) Nodular fasciitis with prominent osteoclast‐like giant cells is a neoplastic lesion, composed of proliferative myofibroblasts with no obvious pleomorphism and atypia, and can be seen mitotic image, but there is no pathological mitotic image. In some cases, osteoclast‐like multinucleated giant cells can be seen in varying degrees and scattered in distribution, but the volume of multinucleated giant cells is relatively small.^5^

Leiomyosarcoma with a large number of osteoclast‐like giant cells is highly malignant. Most LMS‐OGC patients followed‐up in the literature had recurrence or metastasis within a short period of time.^2^, ^6^, ^7^ In the case reported here, leiomyosarcoma differentiation and osteoclast‐like giant cell reaction were found in the phyllodes tumors in the breast, and recurrence occurred nine months after resection. LMS‐OGC is a soft tissue malignant tumor which develops extremely rarely in the breast and should be differentiated and diagnosed according to the history, histological characteristics and immunohistochemical staining.

## Disclosure

The authors declare that there are no conflict of interests.

## References

[1] Darby AJ , Papadaki L , Beilby JO . An unusual leiomyosarcoma of the uterus containing osteoclast‐like giant cells. Cancer 1975; 36: 495–504. 50874 10.1002/1097-0142(197508)36:2<495::aid-cncr2820360228>3.0.co;2-i

[2] Sukpan K , Khunamornpong S , Suprasert P , Siriaunkgul S . Leiomyosarcoma with osteoclast‐like giant cells of the uterus: A case report and literature review. J Med Assoc Thai 2010; 93: 510–5. 20462098

[3] van Meurs HS , Dieles JJ , Stel HV . A uterine leiomyoma in which a leiomyosarcoma with osteoclast‐like giant cells and a metastasis of a ductal breast carcinoma are present. Ann Diagn Pathol 2012; 16: 67–70. 21216642 10.1016/j.anndiagpath.2010.11.010

[4] Zöller J , Born IA , Manke HG , Drommer RB . Clinical aspects and pathology of malignant fibrous histiocytoma. Fortschr Kiefer Gesichtschir 1988; 33: 25–9. 2842244

[5] Nawar NA , Olsen J , Jelic TM , He C . Primary urinary bladder angiosarcoma with osteoclast‐like multinucleated giant cells: A case report and literature review. J Case Rep Am 2016; 17: 143–9. 10.12659/AJCR.896266 PMC4784551 26947436

[6] Sasaki T , Kawashima H , Ariizumi T e a . Denosumab as a potential therapeutic option for leiomyosarcoma with osteoclast‐like giant cells: A case report. Mol Clin Oncol 2018; 8: 30–3. 29387393 10.3892/mco.2017.1489 PMC5769267

[7] Ben SR , Mekni A , Oueslati H , Zitouna M , Bouchoucha S . Leiomyosarcoma of the cervix uteri with osteoclast‐like giant cells. Tunis Med 2012; 90: 896–7. 23247793

